# Update on the natural orifice transluminal endoscopic surgery for gallbladder preserving gallstones therapy: A review

**DOI:** 10.1097/MD.0000000000031810

**Published:** 2022-11-18

**Authors:** Lifeng Shang, Xin Shen, Wenkai Niu, Yi Zhang, Junwei Han, Haiwang Liu, Lei Liu, Xinli Chen, Yiyue Zhang, Shi Hai

**Affiliations:** a Department of Gastrointestinal Surgery, Xi’an Daxing Hospital, Xi’an, P.R. China.

**Keywords:** cholecystolithotomy, flexible endoscopy, gallbladder preservation, gallstones, minimally invasive surgery, natural orifice transluminal endoscopic surgery

## Abstract

Cholecystectomy remains the “gold standard” for the management of symptomatic gallstones. Minimally invasive laparoscopic cholecystectomy has been the treatment of choice for the past 3 decades. However, the technique of natural orifice transluminal endoscopic surgery cholecystolithotomy is evolving, with some experts advocating gallbladder stone removal without gallbladder excision in order to preserve gallbladder function and eliminate post-cholecystectomy syndromes, including complications of the surgical incision, bile duct injury, functional gastrointestinal, and psychological conditions, and possibly an increase in colon cancer. In addition, transluminal endoscopic cholecystolithotomy is an option for elderly patients who are not suitable candidates for open surgery and those who desire scar-free minimally invasive surgery with organ preservation. This article summarizes the established pure natural orifice transluminal endoscopic surgery gallbladder preserving gallstone removal techniques and highlights the pros and cons of different popular available endoscopic approaches to gallstone therapy and how flexible endoscopic surgery via the natural orifice is compared to the well-established cholecystectomy.

## 1. Introduction

Gallstones are one of the most common digestive health problems, with a worldwide prevalence of 10% to 15%.^[1,2]^ Cholecystectomy has remained the first-line treatment since the first open cholecystectomy (in 1882) was performed by Carl Johann August Langenbuch in Berlin.^[3]^ A century later, in 1985, Med Erich Müh performed the first laparoscopic cholecystectomy (LC) ushered in a new age of cholecystectomy with minimal trauma, resulting in lower morbidity, shorter length of hospitalization, and less post-operative pain.^[4]^ This approach also created a precedent for minimally invasive surgery. LC has become the standard treatment for symptomatic cholelithiasis.^[5]^ However, there are groups of patients remaining for which LC may not be the best option, such as elderly people, those with coexisting illness, and those with difficult anatomy of the biliary system (e.g., severe inflammatory adhesions and no-go Calot’s triangle).^[6]^ It is also now recognized that the gallbladder has an indispensable function in regulating bile flow and storing bile that is disrupted by cholecystectomy. LC is not without problems and complications, including post-cholecystectomy syndromes, surgical incision complications, and bile duct injury.^[7–10]^ Cholecystectomy has also been associated with an increased risk of colon cancer and functional gastrointestinal and psychological conditions, and these concerns are listed among the reasons for gallbladder preservation.^[11–13]^ Recently, a novel alternative technique of natural orifice transluminal endoscopic surgery (NOTES) cholecystolithotomy without gallbladder excision has evolving.^[14–17]^ This approach includes pure NOTES trans-rectal/trans-gastric gallbladder preserving cholecystolithotomy and endoscopic ultrasonography-guided gallbladder-preserving cholecystolithotomy.

This article aims to summarize the established pure NOTES gallbladder preserving cholecystolithotomy and highlights the pros and cons of different popular available endoscopic approaches to gallstone therapy and how flexible endoscopic surgery via the natural orifice is compared to the well-established cholecystectomy. In addition, our article highlights areas of future research while stressing on the need for multinational collaboration to provide the steppingstone(s) needed to bring NOTES to the mainstream.

## 2. Technical obstacles in NOTES adoption

NOTES involves insertion of a flexible endoscope into the human body through a natural lumen (such as the mouth, anus, and vagina). This flexible endoscopic-assisted operation via a natural orifice overcomes the disadvantages of rigid instrument-assisted laparoscopic procedures, including minimal damage, cosmetic considerations, post-operative pain, and most importantly, preservation of the organ. Experimental studies on pure NOTES have proposed procedures as attractive alternative options for intra-abdominal procedures.^[18–20]^ However, some technical obstacles remained including the risk of fecal contamination and peritoneal infection, availability of methods for safe entrance into the peritoneal cavity as well as for reliable entry site closure.^[21,22]^ A recent study confirmed the efficacy of using a detachable endo-colonic balloon to keep the distal colonic and rectal lumen clean and aseptic during transrectal NOTES endoscopic procedures.^[23]^ However, successful clinical application requires overcoming these challenges before the widespread adoption of NOTES.

## 3. Description of NOTES gallbladder preserving cholecystolithotomy techniques

Trans-rectal access: In 2015, Liu et al^[24]^ reported a trans-rectal NOTES procedure for gallbladder preserving cholecystolithotomy. The main steps include: routine bowel cleansing, placement of a detachable colonic balloon to prevent peritoneal contamination, incision on the anterior rectal wall, endoscope insertion into the peritoneal cavity via trans-rectal incision, locating and opening the gallbladder wall, extraction of gallstones, irrigation of the peritoneal cavity to avoid peritoneal infection, and closure of the gallbladder incision using endoclips and rectal stoma with endoclips and endoloops.

Trans-gastric access: In 2020, Li and Han and in 2022, Zhang et al reported the transgastric NOTES procedure.^[25,26]^ The main steps for transgastric NOTES cholecystolithotomy include stomach cleansing with sterile saline solution, insertion of a disinfected gastroscope with a transparent cap attached on the tip of the endoscope and a longitudinal incision on the greater curvature with Hook/IT knives, and endoscope entry into the peritoneal cavity. The rest of the steps were similar to that of transrectal NOTES.

Endoscopic ultrasonography (EUS) transgastric luminal apposing metal stent (LAMS) method: This is a stent-applied intervention in which a fistula is produced that bridges the gastrointestinal tract and gallbladder.^[27,28]^ The LAMS is placed along the guidewire, assisted by an electrocautery-enhanced delivery system. LAMS can shorten the distance between the gallbladder and gastrointestinal tract wall. Oral cholecystoscopy can be performed when a mature fistula is formed. The endoscope was advanced into the gallbladder cavity via the fistula formed by the LAMS. A stone retrieval basket is introduced into the gallbladder and gallstones can be discharged into the gastrointestinal tract with the assistance of water flushing. Endoscopic holmium laser lithotripsy is necessary for the treatment of large gallstones. The LAMS can be removed after clearing the stones with a snare or grasping forceps, and the fistula is closed using endoclips.

## 4. NOTES gallbladder preserving cholecystolithotomy—indications and patient selection

Based on the current experience, pure NOTES trans-rectal/trans-gastric gallbladder preserving cholecystolithotomy and endoscopic ultrasonography-guided gallbladder-preserving cholecystolithotomy may be suitable for specific patients who are not candidates for cholecystectomy, including severe acute cholecystitis and high-risk surgical patients and difficult anatomy of the biliary system; and elderly patients not suitable candidates for surgery; patients strongly desired to preserve their organs, and patients with post-operative scarring were mainly women.

## 5. Trans-rectal vs. Trans-gastric route for NOTES

In our experience, both approaches have their advantages and disadvantages. The advantages of transrectal NOTES include easy access to the upper abdomen, especially the gallbladder, and early diet intake (e.g., 6 hours after the procedure, patients are able to consume a liquid diet). For transrectal NOTES, the patient is placed in the supine position, which allows one to wash the peritoneal cavity completely, thus reducing the risk of peritonitis, and less post-operative pain, the problem with this approach is the requirement for bowel preparation and disinfection before the procedure.

The advantage of transgastric NOTES is the lack of requirement for preoperative bowel cleansing. Disadvantages includes: more difficult in accessing the gallbladder and performing the incision and extracting the stones. The fluid intake is delayed, and a drainage tube may be required. Difficulty in stabilizing the endoscope. The patient’s position is left lateral, which makes it difficult to wash completely in the event of bile leakage, possibly increasing the risk of peritonitis, and closing the stomach stoma is more difficult than closing the rectal stoma due to a thick gastric wall. NOTES cholecystolithotomy also avoids surgical scarring, which is very important in some patients.

## 6. Past, present and future of gallbladder-preserving NOTES cholecystolithotomy

Although cholecystectomy remains the mainstay for gallstone treatment owing to its unique merits, it may be frustrated in surgical patients with high risks or biliary deformity. Gallbladder-preserving NOTES cholecystolithotomy has been increasingly offered as an alternative approach for the treatment of symptomatic gallstones, especially in China. The justification for this practice includes considerations regarding safety and post-operative pain, patients unfit for surgery, especially the elderly, and cosmetic results and patient satisfaction. Additionally, some patients with specific cultural backgrounds prefer not to undergo organ removal. Chinese people have been strongly influenced by traditional Chinese culture and traditional Chinese medicine and do not casually undergo the operative removal of organs.^[29]^ In addition, in clinical practice, we found that many patients who undergo operative treatment for cholelithiasis express a strong desire for preservation of the gallbladder.

NOTES cholecystolithotomy is a new procedure and its experience is largely limited to a few units. Importantly, long-term follow-up data on stone recurrence, cholecystitis, abdominal adhesions, and gallbladder dysfunction remain scarce. It has been manifested that the success rate of EUS trans-gastric LAMS is more than 95% and the adverse rate is less than 10%.^[27]^ Similar to pure NOTES transrectal gallbladder preserving cholecystolithotomy, the potential risks of EUS-assisted gallbladder preserving therapy require further investigation through the accumulation of more long-term follow-up data (e.g., stone recurrence, cholecystitis, abdominal adhesions, and gallbladder dysfunction). Gallstone recurrence remains a concern, a recent report showed the average recurrence risk was 3% in 4 years and 10% in 15 years.^[30,31]^ Ullah et al^[32]^ published a propensity score-matched retrospective comparative study of NOTES and LC for the management of gallstones. Successful stone removal was achieved in 86 of the 87 (98.9%) patients in the NOTES arm. Within a median follow-up of 12 months (range: 6–40 months), gallstones and cholecystitis recurred in 10.5% and 3.5% of the patients, respectively. The widespread use of NOTES cholecystolithotomy may require the development of a reliable method to prevent the recurrence of gallstones.

NOTES faced many challenges, and in some experts’ views, it was going to die long ago. Laparoscopic surgery itself in the beginning had many controversies and was not accepted by the majority of surgeons if not all of them. However, laparoscopic surgery has become popular and has been performed (practiced) as a routine procedure. NOTES is also in its early stages, and we believe that it has a good future. Many facts are known by practice and there is Chinese saying which means “practice is the only standard to test the truth.” Many studies have shown that NOTES is a feasible alternative for the management of patients with symptomatic gallstones. Its advantages include no skin wounds, organ retention, quick recovery, fewer post-operative complications, and patient satisfaction. Although this procedure is unlikely to replace LC, it proved useful for most of the patients (especially the elderly or patients incompatible for surgery due to coexisting illnesses and those desiring to retain organs). In this review, we discussed the initial results with the fellow gastroenterologists and surgeons. It is important to report early results so that as others attempt similar experiments they will know what to expect and plan to overcome the weakness.

## 7. Conclusion

We agree that some doctors are pushing the limits, and like in LC, there will be more time before NOTES is more common. The issue is to define the selected group of patients that benefit from NOTES the most and exclude the selected patients that should not be wrongly treated by NOTES for cosmetics, misconceptions, or myths. This must be considered experimental at best and should be restricted in clinical trials under carefully monitored conditions with long-term follow-up. We need more data on this issue before firm recommendations can be made.

## Author contributions

**Conceptualization:** Lifeng Shang.

**Data curation:** Xin Shen, Lei Liu.

**Formal analysis:** Xin Shen, Lei Liu.

**Investigation:** Junwei Han, Lei Liu.

**Methodology:** Wenkai Niu, Xinli Chen, Yiyue Zhang.

**Project administration:** Shi Hai.

**Resources:** Wenkai Niu, Haiwang Liu.

**Validation:** Haiwang Liu.

**Visualization:** Haiwang Liu.

**Writing – original draft:** Lifeng Shang, Xin Shen.

**Writing – review & editing:** Yi Zhang, Shi Hai.
